# Frequency of Early Morbidities in Low Birth Weight Neonates at The Aga Khan University Hospital, Karachi

**DOI:** 10.7759/cureus.6061

**Published:** 2019-11-03

**Authors:** Saroop Chand, Fayaz Ahmed, Muhammad Hussain Shah, Abdul Lateef Leghari, Parveen Usman, Rohan Advani, Muhammad Sohail Salat, Shabina Ariff

**Affiliations:** 1 Pediatrics and Child Health, Aga Khan University Hospital, Karachi, PAK; 2 Pediatrics, Aga Khan University Hospital, Karachi, PAK; 3 Pediatrics, Aga Khan University, Karachi, PAK

**Keywords:** low birth weight, prematurity, early morbidities, neonates, respiratory distress syndrome, hypoglycemia, hypothermia, hypocalcemia, nicu

## Abstract

Background

Globally, approximately 14.6% children are born with low birth weight (LBW) annually. In Pakistan, this figure however reaches approximately 16%. Low birth weight infants are vulnerable to develop early morbidities like hypothermia, hypoglycemia, respiratory distress syndrome and hypocalcemia. There is a scarcity of statistics which creates a gap in development of strategies for improving quality of care in developing countries. The aim of our study was to determine the frequency of early morbidities such as respiratory distress syndrome (RDS), hypoglycemia, hypothermia and hypocalcemia in low birth weight neonates.

Methodology

A prospective descriptive study was conducted via non-probability sampling technique from 1^st^ April 2016 to 30^th^ September 2016 at The Aga Khan University Hospital, Karachi. All low birth weight infants, i.e., those with birth weight < 2500 grams were included in this study and observed for early morbidities, including hypothermia, hypoglycemia, hypocalcemia and respiratory distress syndrome. Descriptive analysis was done using SPSS version 22 (IBM Corp., Armonk, NY), mean and standard deviation were determined for quantitative variables, whereas frequency and percentages were calculated for qualitative variables.

Results

A total of 2082 neonates were born during the study period, of which 271 (13%) were born with low birth weight. One hundred and eighty-five (68.1%) of these LBW neonates were preterm babies while 86 (31.9%) were born at term. Among LBW neonates 137 (51.0%) were males and 134 (49.0%) females. In the study population, hypoglycemia was seen in 17.3%, hypocalcemia in 13.6%, respiratory distress syndrome in 11%, and hypothermia in 2.5%.

Conclusion

Our study highlighted major early morbidities of LBW neonates, and their association with birth weight, gestational age and gender. Significant association of birth weight was found with hypothermia and hypocalcemia, whereas hypocalcemia and RDS were significantly associated with gestational age. However, none of the early morbidities had significant association with gender. Keeping in perspective the early morbidities in this population we propose that priority be given to providing adequate attention to low birth weight neonates.

## Introduction

Newborns with birth weight less than 2500 grams are called low birth weight (LBW) [1]. Neonatal mortality and morbidity are an alarming public health issue in most developing countries. The neonatal period ranks the highest risks of death among any period in the human lifetime [2]. In developed world, neonatal health reforms have gained focus in child health for the reduction of both morbidity and mortality. In developing countries however, strategies for improving neonatal health standards have not received significant attention.

The United Nations Children's Fund (UNICEF) estimates show that globally approximately 20 million children are born with LBW annually. LBW neonates prevalence is much higher in the developing countries (16.5%) compared to developed countries (7%); with the highest incidence in south central Asia (27%) followed by Africa (13%-15%) [1,3]. Burden of LBW among neonates in Pakistan is approximately 16%. LBW is predominantly due to prematurity and/or intra-uterine growth restriction (IUGR) [4], however in developing countries it is most commonly due to IUGR rather than prematurity [3-5]. Prematurity and IUGR are associated with increased neonatal morbidity and mortality [6].

Low birth weight IUGR newborns are prone to develop early morbidities such as birth asphyxia, hypothermia, hypoglycemia, hypocalcemia and polycythemia [7,8], whereas preterm low birth weight neonates suffer from hypothermia, respiratory distress syndrome, apnea, hypoglycemia, seizures, jaundice, feeding difficulties, periventricular leukomalacia [9,10].

Low birth weight neonates often develop hypoglycemia, evident by study conducted on neonatal hypoglycemia in babies identified as high risk, which reported a 27% incidence of hypoglycemia in low birth weight neonates [11]. In a study conducted in Shimla hospital, India regarding neonatal morbidity, 6.3% developed varying degrees of birth asphyxia, 3.9% developed respiratory distress syndrome, 2.9% developed hypothermia [12]. In a study conducted on hypocalcemia in low birth weight neonates, early neonatal hypocalcemia was observed in approximately 24% neonates [13]. The purpose of this study was to determine the frequency of early morbidities in LBW neonates regardless of gestational age such as hypothermia, hypoglycemia, respiratory distress syndrome (RDS) and hypocalcemia.

Limited literature is available from developing countries in this regard, which hinders development and implementation of new strategies for improving quality of care in developing countries. Any step towards establishing such strategies is dependent on availability of data on early morbidities in low birth weight neonates, which can help in determining the neonatal health status at national level.

## Materials and methods

This prospective descriptive study was conducted at Neonatal Intensive Care Unit (NICU) and Well Baby Nursery of The Aga Khan University Hospital, Karachi from 1st April 2016 to 30th September 2016, after approval from the hospital ethical review committee. Our NICU is a Level III NICU with state-of-the-art facilities and a capacity of 24 beds. Neonates after appropriate management in NICU are shifted to mother baby step down unit. A total of 271 neonates born with low birth weight (<2500 grams) were enrolled by non-probability consecutive sampling technique, out of 2082 neonates born at our institute during the study period. Inclusion criterion was neonates with birth weight <2500 grams regardless of gestational age. Exclusion criteria were neonate with any congenital anomalies, discharged before 72 hours of life, those who underwent any surgical procedure. All neonates who fulfilled these criteria were selected for the study after taking informed consent from their parents. Data was collected on a structured proforma which included demographics as well as variables under study including hypothermia, hypoglycemia, RDS and hypocalcemia, for which all neonates were monitored closely. Neonates were monitored during the initial 72 hours for early morbidities. Descriptive analysis was carried out using SPSS version 22 (IBM Corp., Armonk, NY). Mean and standard deviations were calculated for birth weight. Frequency and percentage were calculated for early morbidities such as hypoglycemia, hypothermia, respiratory distress, and hypocalcemia. Chi-square test was conducted to understand the impact of these on result variables. p value ≤0.05 was taken as significant.

## Results

During the study period, a total of 2082 neonates were born out of which 271 newborn babies fulfilled the criteria. Male to female ratio among these neonates was almost equal with 137 (51%) being males while the rest 134 (49%) were females. Among these neonates 185 (68.26%) were preterm while 86 (31.7%) were term. The mean birth weight of enrolled neonates was 2.055 kilograms ± 0.229 SD and mean gestational age was 35.2 weeks ± 1.99 SD. The frequency of the morbidities under study was as follows: morbidities were present in 44.6% (n = 121) low birth weight neonates, RDS in 11% (n = 30), hypoglycemia in 17.3% (n = 47), hypocalcemia in 13.6% (n = 37), hypothermia in 2.5% (n = 7), while 56.4% (n = 150) LBW neonates had no early morbidities (Table 1). Risk estimation of the morbidity was done with gestational age, gender and birth weight by using Chi square test (confidence interval 95%) and it was found that there was no gender predominance as far as the early morbidities were concerned (p-value > 0.05) but significant association with gestational age (p-value < 0.05) was present. Similarly, the morbidities significantly declined with the increase in birth weight among the low birth weight neonates.

**Table 1:** Demographics and Frequency of Early Morbidities in Low Birth Weight Neonates. RDS: Respiratory distress syndrome

Variable	Frequency	Percentage
Gender		
Male	137	51%
Female	134	49%
Gestation		
Preterm	185	69%
Term	86	31%
Birth weight		
<1500 grams	121	44.60%
1500-<2500 grams	150	55.40%
Early morbidities		
Hypoglycemia	47	17.3%
RDS	30	11%
Hypocalcemia	37	13.6%
Hypothermia	7	2.5%
No early morbidity	150	56.4%

According to Table 2, hypoglycemia was present in 21 (15.3%) of the males and 26 (19.4%) of the females. In regard to gestational age hypoglycemia was present in nine (19.6%) of neonates born at <34 weeks and in 38 (16.9%) of neonates born at ≥34 weeks gestation. Trends in birth weight also show a rise in occurrence of hypoglycemia with decreasing birth weight.

**Table 2:** Association of Hypoglycemia with Gender, Gestational Age and Birth Weight.

	Hypoglycemia				Total	P Value
	Yes		No			
	Frequency	Percentage	Frequency	Percentage		
Gender						
Male	21	15.3%	116	84.7%	137	0.186
Female	26	19.4%	108	80.6%	134	
Gestational age (Weeks)						
<34	9	19.6%	37	80.4%	46	0.061
≥34	38	16.9%	187	83.1%	225	
Birth weight (kgs)						
<1.5	35	29.7%	83	70.3%	118	0.058
1.5-<2.5	12	7.8%	141	92.2%	153	

According to Table 3, respiratory distress syndrome was present in 19 (13.9%) of the males and 11 (8.2%) of the females. In regard to gestational age RDS was present in 15 (32.6%) of neonates born at <34 weeks and in 15 (6.7%) of neonates born at ≥34 weeks gestation, and was statistically significant with p value < 0.001. Trends in birth weight show a rise in occurrence of RDS with decreasing birth weight.

**Table 3:** Association of RDS with Gender, Gestational Age and Birth Weight. RDS: Respiratory distress syndrome

	RDS				Total	P Value
	Yes		No			
	Frequency	Percentage	Frequency	Percentage		
Gender						
Male	19	13.9%	118	86.1%	137	0.186
Female	11	8.2%	123	91.8%	134	
Gestational age (Weeks)						
<34	15	32.6%	31	67.4%	46	<0.001
≥34	15	6.7%	210	93.3%	225	
Birth weight (kgs)						
<1.5	24	20.2%	95	79.8%	119	0.111
1.5-<2.5	6	3.9%	146	96.1%	152	

According to Table 4, hypocalcemia was present in 21 (15.3%) of the males and 16 (11.9%) of the females. In regard to gestational age hypocalcemia was present in 13 (28.3%) of neonates born at <34 weeks and in 24 (10.7%) of neonates born at ≥34 weeks gestation, and was statistically significant with p value < 0.001. In our study birth weight and hypocalcemia had significant association (p-value < 0.05) and showed a decrease in occurrence of hypocalcemia with increase in birth weight.

**Table 4:** Association of Hypocalcemia with Gender, Gestational Age and Birth Weight.

	Hypocalcemia				Total	P Value
	Yes		No			
	Frequency	Percentage	Frequency	Percentage		
Gender						
Male	21	15.3%	116	84.7%	137	0.263
Female	16	11.9%	118	88.1%	134	
Gestational age (Weeks)						
<34	13	28.3%	33	71.7%	46	<0.001
≥34	24	10.7%	201	89.3%	225	
Birth weight (kgs)						
<1.5	26	22.0%	92	78.0%	118	0.009
1.5-<2.5	11	7.2%	142	92.8%	153	

According to Table 5, hypothermia was present in one (0.7%) of the males and six (4.5%) of the females. In regard to gestational age hypothermia was present in one (2.2%) of neonates born at <34 weeks and in six (2.7%) of neonates born at ≥34 weeks gestation, which was not statistically significant. In our study birth weight and hypothermia had significant association (p-value < 0.05) and showed a decrease in occurrence of hypothermia with increase in birth weight.

**Table 5:** Association of Hypothermia with Gender, Gestational Age and Birth Weight.

	Hypothermia				Total	P Value
	Yes		No			
	Frequency	Percentage	Frequency	Percentage		
Gender						
Male	1	0.7%	136	99.3%	137	0.57
Female	6	4.5%	128	95.5%	134	
Gestational age (Weeks)						
<34	1	2.2%	45	97.8%	46	0.41
≥34	6	2.7%	219	97.3%	225	
Birth weight (kgs)						
<1.5	6	5.1%	112	94.9%	118	0.029
1.5-<2.5	1	0.7%	152	99.3%	153	

## Discussion

Neonatal morbidity and mortality are a critical public health issue across many developing countries. Among multitude of factors, prematurity and low birth weight are major predictors of morbidity and mortality. Despite attempts at devising strategies for improving health status and preventing diseases, little has been achieved in terms of decrease in neonatal morbidity and mortality across various parts of the world. The causes attributing to the consistently increased neonatal mortality rate (NMR) are antenatal, perinatal and postnatal factors. Present study reflects that there was a gradual decline in morbidity with increasing gestational age and birth weight. Therefore, measures such as provision of adequate antenatal and postnatal care, as well as prevention of complications in pregnancy to achieve adequate weight at birth, as well as to prevent preterm delivery are factors that are significantly important in reducing neonatal morbidity.

The frequency of morbidity and mortality among neonates reflects the quality of antenatal, intrapartum and postnatal care, and varies across different institutes and even countries. In developing countries with limited facilities, the survival of high-risk neonates tends to be low, in particular due to inability to address early morbidities in neonatal period. Early morbidities observed in our study were also found in a study conducted by Basu et al., which showed that common morbidities of low birth weight included RDS, apnea, sepsis, hypothermia, hypoglycemia, shock and intraventricular hemorrhage (IVH) [14].

Frequency of hypoglycemia has a wide variability across literature. A study conducted on low birth weight infants found that hypoglycemia occurred in 24% of infants, however, in our study we observed that 17.3% of low birth weight neonates had episodes of hypoglycemia [15]. According to a study conducted in India, 8.4% of LBW neonates developed hypothermia, while in our study we found that hypothermia occurred in 2.5% of low birth weight neonates, with no significant associations with gender and gestational age, however, birth weight and hypothermia had significant association (p-value < 0.05) [12]. In respiratory distress syndrome occurring in this population no significant association was found with birth weight, and literature seems to be very divided on it. However, different studies on RDS have observed that it has no significant association with gender, a finding consistent with our study [16]. There was a positive correlation between gestational age and RDS, which too was consistent with our study [17]. A study on low birth weight neonates found the frequency of hypocalcemia to be 24.4%, our study however found a frequency of 13.6% [13]. There was also significant association of hypoglycemia with gestational age and birth weight.

## Conclusions

Our study highlighted major early morbidities of LBW neonates, and their association with birth weight, gestational age and gender. Significant association of birth weight was found with hypothermia and hypocalcemia, whereas hypocalcemia and RDS were significantly associated with gestational age. However, none of the early morbidities had significant association with gender. In order to minimize early morbidities in low birth weight neonates, adequate consideration should be given to this high-risk population by the health care providers. The data acquired through this study will subsequently be used not only in creating awareness among medical practitioners dealing with neonates, but also in developing protocols for appropriate management and prevention of such morbidities.
